# Chronic respiratory symptoms, lung function and associated factors among flour mill factory workers in Hawassa city, southern Ethiopia: “comparative cross-sectional study”

**DOI:** 10.1186/s12889-020-08950-9

**Published:** 2020-06-11

**Authors:** Zemachu Ashuro Lagiso, Worku Tefera Mekonnen, Samson Wakuma Abaya, Abera Kumie Takele, Hailemichael Mulugeta Workneh

**Affiliations:** 1 College of Health Science and Medicine, Dilla University, P.O. Box 419, Dilla, Ethiopia; 2 Department of Preventive Medicine, School of Public Health, Addis Ababa University, P.O. Box 90861000, Addis Ababa, Ethiopia; 3 College of Health Science, Debre Berhan University, Debre Berhan, Ethiopia

**Keywords:** Flour mill, Respiratory symptoms, Lung function

## Abstract

**Background:**

Occupational related respiratory diseases arise as a result of the deposition of dust particles in the lungs. Flour milling industries; generate organic dust during industrial processes, such as cleaning, milling, packaging, and loading which release dust into the air and later inhaled by workers. Flour mill workers are at risk of developing respiratory health problems because of exposure in their working environment, but existing data were few. The aim of this study was to assess the prevalence of chronic respiratory symptoms, lung function and associated factors among flour mill factory workers.

**Methods:**

A comparative cross-sectional study was conducted among 196 flour mill factory workers and 210 soft drinks factory workers. We selected study participants using a systematic sampling technique. We assessed the chronic respiratory symptoms using the questionnaire adopted from the British Medical Research Council. Binary logistic regression analysis with 95% CI and p < 0.05 was used to identify the factors. Lung function parameters; Forced Vital Capacity (FVC), Forced Expiratory Volume in one second (FEV1) and ratio FEV1/FVC was measured by using spirometer and analyzed by using an independent t-test.

**Results:**

We included 406 (96.7%) workers in this study. The prevalence of chronic respiratory symptoms was higher among flour mill workers as compared to soft-drinks factory workers (56.6% vs.12.9%). Primary education (AOR = 5.8, 95% CI, 1.3–23.2), mixing department (AOR = 5.3, 95% CI = 1.68–16.56), work experience 6–9 years (AOR = 5.1, 95% CI = 2.05–12.48), work experience ≥10 years (AOR = 2.5, 95% CI = 1.01–6.11) and working over eight hours (AOR = 2.4, 95% CI, 1.16–5.10) were factors that significantly associated with chronic respiratory symptoms among flour mill workers. FVC (p < 0.002), FEV1 (p < 0.001) and FEV1/FVC (p < 0.012) were significantly reduced among flour mill workers.

**Conclusions:**

We found chronic respiratory symptoms to be high among flour mill workers. Lower education level, mixing department, increased work experience, and longer working hours were identified factors. The flour mill dust exposed worker’s lung function parameters were highly reduced. This study suggested that workers’ dust exposure reduction and control methods in flour mill factories need to be implemented.

## Background

Occupational related respiratory diseases arise because of the deposition of dust in the lungs. The deposited dust disturbs the process of inhalation and exhalation of air whose intensity is influenced by the dust, duration of exposure, concentration, and size of dust in the breathing zone and genetic factors [1, 2]. Chronic respiratory diseases comprise 10% of all occupational diseases reported in industrialized countries like the United Kingdom but much higher in the rapidly developing country like Nigeria [3, 4].

Flour is a complex organic dust containing powder finished production of milling cereals like wheat, barley, corn (maize), or a combination of these, got after several reduction processes [5]. Flour milling industries generate organic dust during industrial processes, such as cleaning, milling, packaging, and loading which releases dust into the air and later inhaled by workers [6]. Vulnerability to flour dust in the workplace becomes high during mixing and packing in the flour milling process and had high respiratory symptoms [7]. A dusty environment and unfavorable climatic conditions are major occupational hazards in a flour factory that influence the health of workers. Dust generated during flour production has many hazardous substances include silica, fungi, their metabolites, and endotoxin, which causes respiratory disease [1, 8].

Flour mill workers are at risk of developing respiratory health effects due to exposure in their working environment [9]. Lung function tests are useful in assessing the functional status of the respiratory system and based on the measurement of the volume of air breathed in and out during quiet breathing and forced [10]. Spirometry is one of the most important diagnostic tools in the diagnosis of respiratory diseases and describes the effects of lung function [11].

Flour dust exposure causes respiratory symptoms such as chest pain, phlegm, wheezing, cough, and shortness of breathing in flour mill workers [7, 12–14]. In addition, studies conducted in Saudi Arabia, Belgium, Iran, UK and Ethiopia found that a reduction of lung function parameters; Forced Expiratory Volume in 1  second (FEV_1_), Forced Vital Capacity (FVC) and the ratio of Forced Expiratory Volume in 1  second and Forced Vital Capacity (FEV_1_/FVC) among flour factory workers [12, 15–18].

Study conducted in 1998 in Ethiopia reported that a higher prevalence of respiratory symptoms like a cough (42.1%), wheezing (35.7%) and chest tightness (35.1%) in flour mill workers but did not investigate their associated factors and lung function [18, 19]. The most recent study conducted in Addis Ababa flour mill workers investigated both prevalence of respiratory symptoms and lung function but didn’t investigate associated factors [18]. Therefore, this study was conducted to assess chronic respiratory symptoms, lung function and associated factors among flour mill workers in Hawassa city, Southern Ethiopia.

## Methods

### Study design and setting

An institutional-based comparative cross-sectional study was conducted in April 2018 among flour mill and soft drink factory workers in Hawassa city of Southern Ethiopia. In Hawassa city, there are ten flour processing factories (4 medium scales and 6 small scales) and one soft drink factory.

### Study population

One hundred ninety-six flour mill workers worked for at least one year in the factory were included in this study. In addition, we included two hundred ten soft drinks factory workers as a comparison group. We performed a lung function test only for 108 randomly selected workers (54 from the flour mill factory and 54 from the soft drink factory) from 406 study participants because of limited resources.

### Inclusion and exclusion criteria

We included all workers above 18 years of age who had direct involvement in production and working in the flour mill and soft drink factory for one year and above. Workers who had heart failure, tuberculosis, drug addicts, cigarette smokers, emphysema, recent surgery of the thorax, abdomen, undergone vigorous exercise and any acute illness were excluded from the lung function test.

### Sampling procedures

First, the work process was stratified by production units in both flour mill and soft drink factories. Then, systematic sampling techniques applied to draw the required sample size. The workers’ roster was used as a sampling frame. First subject selected randomly from each department, then we selected other subjects every 2^nd^ interval by taking roster of the workers in each department.

### Variable measurement

#### Outcome variables

Chronic respiratory symptoms: are defined as the development of one or more of the respiratory symptom(s) of a chronic cough, chronic cough with sputum, chronic breathlessness, chronic wheezing, chronic chest illness which lasts at least three months in one year [20, 21].

Lung function reduction: Forced vital capacity (FVC); the maximum volume of air that can be breathed out forcefully and rapidly following a maximum inspiration, Forced expiratory volume in 1 second (FEV_1_) (the volume exhaled during the first second of the FVC maneuver) and FEV_1_/FVC; the percentage of the FVC expired in the first second of maximal forced expiration following full inspiration [22].

### Exposure variables

The socio-demographic factors of the workers; age (20–29 years, 30–39 years, 40–49 years, 50–59 years and ≥ 60 years), sex (male, female), educational status (illiterate, primary education, secondary education, certificate and above) and monthly income (20 US$-70 US$ and ≥ 70 US$) were included.

The behavioral factors of workers (like a smoking habit); current smokers (workers who were smoking at the time of the study or a person who smoke cigarettes every day or some days) and every smoker: a worker who has smoked at least one hundred cigarettes during his/her life, which includes current smokers [23]. The work-related factors included the work experience in the factory (1–5 years, 6–9 and ≥ 10 years), working hours per day (8 h or greater than 8 h) and working departments; Cleaning (the first milling steps involve manually separating wheat from seeds and other grains, Mixing (a mix process (to combine additives and raw materials) was done manually to mixers, and this activity generates a lot of flour dust which is inhaled by the workers, Packaging (Packing of the flour was done manually whereby the workers fill the flour to the bags and this activity generates a lot of flour dust) and loading **(**carrying of flour from the packing areas to the stores or trucks).

Previous exposure history; workers experience in the dusty environment before the current working position. Previous medical history (like the history of Asthma, Chronic bronchitis, Lung cancer and tuberculosis, emphysema, recent surgery of the thorax, abdomen, undergone vigorous exercise and any acute illness) that could be developed before and confirmed by physicians.

### Ethical approval

We conducted the study after having an ethical clearance from the Institutional Review Board of the College of Health Sciences of Addis Ababa University. Before performing measurements, we obtained verbal and written consent from each study participant and participants informed that they have full rights to refuse and discontinue taking part at any point in the study. The study participants with lung function impairments were advised and linked to a health facility.

### Data collection procedure

#### Interviews

Data were collected by using questionnaires adopted from British Medical Research Council (BMRC) [24]. The components of the questionnaires were socio-demographic variables, work-related variables, common chronic respiratory symptoms variables, behavioral factors of workers and past medical history. The questionnaire translated to Amharic retranslated to back English to check its consistency with the original one. Before the interview: a brief explanation given to the participant about the purpose of the study and administered (face to face) for selected flour mill and soft drink factory workers.

### Anthropometric measurements

Weight of study participants were measured by using a standardized electronic weighing machine, with the subjects standing and wearing light clothes and height of the subjects measured with the stadiometer with portable field survey scales. Body mass index (BMI) calculated by using Ndd Medical technologies’ software.

### Spirometry

Ndd Easy on-PC spirometer was used for determining lung function parameters (FVC, FEV1 and FEV1/FVC) among both flour mill and soft drink factory workers according to the American thoracic society recommendation [25]. Before performing the procedure, the subjects had instructed to practice deep inspiration and complete forceful expiration. We performed the spirometer test before work time. By putting a nose clip to prevent air leak through the nose, the subject was initially breathing for a few breaths normally, followed by deep inhalation and forceful expiration of the air fast and forcefully.

We performed all measurements in the sitting position. While doing this maneuver, flow and volume curves inspected on the screen for detecting whether subjects displayed enough effort during inspiration and expiration. Repeatability of FEV1 and FVC parameters were checked and three acceptable maneuvers with repeatable results were taken and we recorded the highest reading of these. Predicted values for lung function tests were not obtained due to lack of reference equation for the Ethiopian population. Trained and certified health professionals selected from different hospitals performed lung function tests. Standard operative procedures (SOPs) were used to improve quality of lung function test. In addition, the volume was measured by using 3 l syringe at the beginning of the test.

### Statistical analysis

Collected data were organized and entered Epi data version 3.1 and we did cleaning to avoid missing values, outliers, and other inconsistencies. For data cleaning, frequency, sort, and list were used. Cleaned data exported to SPSS version 24 for analysis. Descriptive statistics were used to summarize data. Prevalence odds ratio with 95% CI was used to compare the prevalence of chronic respiratory symptoms of a flour mill with soft drink factory workers. Logistic regression analysis used to identify whether exposure variables are significantly associated with outcome variables or not. Thus, variables with p < 0.2 were included in the multivariable analysis by adjusting confounding variables; age, smoking habit, income, educational status, past dust exposure, working hours per day. The P-value of < 0.05 was considered as statistically significant. An independent t-test was used for continuous data; to compare the lung function test between flour mill and soft drink factory workers.

## Results

### Socio-demographic characteristics

Four hundred six (96.7%) workers were included in this study (196 from the flour mill factories and, 210 from the soft drink factory), from 10 flour mill factories and one soft drink factory in Hawassa city, southern Ethiopia. From 420 flour mill workers, 4 workers were refused to take part, 4 workers were on sick leave, and six had stopped working in the factory. Most study participants were males, which accounts for 78.3% and (mean ± SD) age of flour mill workers were 32.1 ± 9.99 and for a soft drink, factory workers were 31.28 ± 7.87 and ranging from 20 to 63 years. Seventy-three (73%) of participants from flour mill factories had attained a primary level of education and about 103 (49%) of soft drink factory participants had attained more than secondary education.

One hundred sixty-three (83.2%) of participants from flour mill factories had a monthly income between 20 US$ and 70 US$ and, 167 (79.5%) of soft drink factory participants ≥70 US$. There was a significant difference between the flour mill factories and soft-drinks factory participants in terms of age, educational status, income and smoking habit (ever smoker) (p < 0.05). In this study, 39 (19.9%) of flour mill factory workers and 4 (1.9%) of soft-drinks factory workers were ever smokers (Table 1).

**Table 1:** Socio-demographic characteristics of flour mills and soft drink factory workers, Hawassa city, South Ethiopia, 2018

Variables	Flour mill factory (n = 196)	Soft drink factory (n = 210)	P-value
	n (%)	n (%)	
Sex of respondents			
Male	157(80.1)	161 (76.7)	0.401
Female	39 (19.9)	49 (23.3)	
Age (in years)			
20–29 years old	100 (51.0)	107 (51.0)	
30–39 years old	66 (33.7)	72 (34.0)	
40–49 years old	8 (4.1)	21 (10.0)	0.295
50–59 years old	15 (7.6)	10 (5.0)	
> = 60 years old	7 (3.6)	1 (1.0)	
Educational status			
Primary education	144 (73.6)	42 (20)	0.0001**
Secondary education	36 (18.4)	65 (31)	
More than secondary	16 (8)	103 (49)	
Monthly income			
20–70 US$	163 (83.2)	43 (20.3)	0.000**
≥ 70 US$	33 (16.8)	167 (79.5)	
Ever smokers			
Yes	39 (19.9)	4 (1.9)	0.000**
No	157 (80.1)	206 (98.1)	

P-value: Statistical significance level: * p < 0.05; **p < 0.01

### Work-related factors

Hundred one (51.5%) of flour mill factory workers and 24 (14.4%) of soft drink factory workers had an experience of dust exposure before joining the present job. From flour mill factory workers about 34% had work experience in the factory for six years or more and about 36% had work experience for over ten years. One hundred forty-five (69%) of the soft-drinks factory and 95 (46.5%) of flour mill factory workers were working greater than 8 h per day. Few flour mill factory workers 11 (5.6%) and soft drink factory workers 9 (4.3%) were reported the chronic respiratory disease confirmed by physicians before they have started a job in the factory. There was a significant difference between flour mill factory workers and soft-drinks factory workers in terms of duration of working hours and past dust exposure, p < 0.05 (Table 2).

**Table 2:** Work related factors and past respiratory illnesses of flour mills and soft drink factory workers, Hawassa city, South Ethiopia, 2018

Variables	Flour mill factory (n = 196)	Soft drinks factory (n = 210)	P-value
	n (%)	n (%)	
Work experience			
1–5 years	60 (30)	83 (39.5)	
6–9 years	66 (34)	69 (32.9)	0.110
≥ 10 years	70 (36)	58 (27.6)	
Working hours per day			
8 h	101 (51.5)	65 (31)	0.013*
> 8 h	95 (48.5)	145 (69)	
Past dust exposure			
Yes	101 (51.5)	24 (11.4)	
No	95 (48.5)	186 (88.6)	0.000**
Energy used at home			
Charcoal	151 (77)	160 (76.2)	
Electricity	45 (23)	50 (23.8)	0.840
Previous respiratory illness			
Yes	11 (5.6)	9 (4.3)	
No	185 (94.4)	201 (95.7)	0.648

P-value: Statistical significance level: * p < 0.05; **p < 0.01

#### Prevalence of chronic respiratory symptoms

By adjusting past dust exposure, working hours per day, educational status, smoking habit and monthly income, the prevalence of respiratory symptoms between flour mill and soft drink factory workers were statistically significant for chronic cough (39.3%; PR = 1.53, 95% CI = 1.53–2.03), chronic cough with sputum (17.86%; PR = 1.34, 95% CI = 1.02–1.76), chronic wheezing (17.35%; PR = 1.33, 95% CI = 1.01–1.75), chronic breathlessness (18.9%; PR = 1.42, 95% CI = 1.08–1.87) and at least one chronic respiratory symptoms (56.6% Vs. 12.86%). The odds of chronic respiratory symptoms in flour mills are 32% more than that of the soft drink factory (PR = 1.32, 95% CI, 1.05–1.65) (Table 3).

**Table 3:** Prevalence of respiratory symptoms in flour mill and soft drink factory workers, Hawassa city, South Ethiopia, 2018

Variables	Flour mill factory (n = 196)	Soft drinks factory (n = 210)	PR (95%CI)
	n (%)	n (%)	
At least one chronic respiratory symptom	111 (56.6)	27 (12.9)	1.32 (1.0–1.6) *
Chronic chough	77 (39.3)	12 (5.7)	1.53 (1.1–2.0) *
Chronic breathlessness	37 (17.9)	18 (5.2)	1.42 (1.1–1.9) *
Chronic cough with sputum	35 (17.9)	9 (4.3)	1.34 (1.0–1.8) *
Chest wheezing	34 (17.3)	8 (3.8)	1.33 (1.0–1.7) *
Chest tightness	23 (11.9)	6 (2.9)	1.20 (0.7–1.9)

*PR* prevalence Ratio, *CI* confidence interval and ^*^p < 0.05, adjusting for past dust exposure, working hours per day, educational status, smoking habit and monthly income

### Factors associated with chronic respiratory symptoms

The multivariate analysis is summarized in (Table 4) educational status, working department, working hours and service years of the workers were significantly associated with chronic respiratory symptoms (p < 0.05).

Workers whose education level was primary were 5.8 times more likely to develop chronic respiratory symptoms than workers whose education level was secondary and above (AOR = 5.8, 95% CI =1.3–23.2). Workers who were engaged in mixing department were 5.3 times more likely to develop chronic respiratory symptoms than workers engaged in cleaning, loading and packaging sections (AOR = 5.3, 95% CI = 1.68–16.56). Workers who had work experience 6–9 years (AOR = 5.1, 95% CI = 2.05–12.48) and work experience ≥10 years (AOR = 2.5, 95% CI = 1.01–6.11) had the odds of developing chronic respiratory symptoms 5.1 and 2.5 times more than workers who had work experiences between 1 and 5 years, respectively. Workers who stay greater than 8 h per day were 2.4 times more likely to develop chronic respiratory symptoms than those who had 8 h or less (AOR = 2.4, 95% CI, (1.16–5.10).

**Table 4:** Respiratory symptoms and associated factors in flour mill factory workers, Hawassa city, South Ethiopia, 2018

Variables	At least one chronic respiratory symptoms		COR (95%CI)	AOR (95%CI)
	Yes	No		
Sex				
Male	95	62	2.2 (1.1–4.5) *	0.84 (0.3–2.8)
Female	16	23	1.00	1.00
Age (in years)				
20–29 years old	48	52	1.00	1.00
30–39 years old	41	25	3.2 (0.9–3.3) *	1.4 (0.6–3.1)
40–49 years old	6	2	3.2 (0.6–16.9) *	1.96 (0.3–13.4)
50–59 years old	11	4	3.0 (0.9–10.0) *	2.5 (0.6–10.7)
≥ 60 years old	5	2	2.7 (0.9–9.9)	2.9 (0.3–31.4)
Educational status				
Primary education	90	54	3.7 (1.2–10.6) *	5.8 (1.3–23.2)**
Secondary education	16	20	1.8 (0.5–6.1)	3.1 (0.7–13.3)
More than secondary	5	11	1.00	1.00
Past dust Exposure				
Yes	66	45	2.1 (1.2–3.7) *	1.5 (0.7–3.2)
No	35	50	1.00	1.00
Working departments				
Cleaning	11	19	1.00	1.00
Loading	37	26	2.5 (1.0–6.0) *	3.1 (0.9–9.6)
Mixing	60	26	4.0 (1.6–9.6) *	5.3 (1.7–16.6) **
Packaging	3	14	0.4 (0.1–1.6)	0.52 (0.1–2.7)
Work experience				
1–5 years	19	41	1.00	1.00
6–9 years	44	26	5.7 (2.7–12.4) *	5.1 (2.1–12.5) **
≥ 10 years	48	18	3.6 (1.8–7.6) *	2.5 (1.0–6.1) **
Working hours per day				
≤ 8 h	37	47	1.00	1.00
> 8 h	74	38	2.5 (1.4–4.4) *	2.4 (1.2–5.1) **

Note: 1.00 = reference value *p < 0.2 for COR **p < 0.05 for AOR, At least one chronic respiratory symptom: defiend as the development of one of the respiratory symptoms

### Anthropometric measurements

Table 5 shows the comparison of the anthropometric parameters between the flour mill workers and soft drink factory workers. We found no significant difference between the groups regarding age, body mass index, weight and height.

**Table 5:** Anthropometric data for flour mill workers compared with soft drink factory workers, Hawassa city, South Ethiopia, 2018

Variables	Flour mill Workers (n = 48) Mean (±SD)	Soft drink factory workers (n = 48) Mean (±SD)	P-value
Age (years)	31.96 ± 9.78	29.58 ± 8.94	0.43
Weight (Kg)	*59.40 ± 7.78*	64.02 ± 8.26	0.24
Height (cm)	*1.67 ± 0.07*	1.69 ± 0.07	0.85
BMI (Kg/m^2^)	21.42 ± 2.04	22.40 ± 2.56	0.06

*P* -Value Significance level: *p < 0.05* “n”: Number of study participants

### Lung function test

We included one hundred eight male workers (54 from the flour mill factory and 54 from the soft drink factory). Six Spirometers results among flour mill workers and six among the soft-drinks factory workers were excluded from the analysis because of unacceptable readings. We present the mean values of the lung function parameters for the total number of flour mill workers and soft in (Table 6). The values of FVC, FEV1 and FEV1/FVC were significantly reduced (p < 0.05) among flour mill factory workers compared to soft-drinks factory workers.

**Table 6:** Lung function test parameters in 48 flour mill and 48 soft drink workers, Hawassa city, South Ethiopia, 2018

Parameters	Flour mill workers (n = 48) Mean (±SD)	Soft-drinks factory workers (n = 48) Mean (±SD)	P-Value
FVC (liters)	*2.78* ± 0.67	3.19 ± 0.59	0.002**
FEV_1_ (liters)	3.46 ± 0.70	3.91 ± 0.65	0.001**
FEV_1_/FVC%	74.10 ± 9.12	79.87 ± 12.54	0.012*

*P* – Value Significance level; * *p* < 0.05; **p < 0.01

## Discussions

The prevalence of almost all chronic respiratory symptoms was higher among flour mill factory workers, compared with soft-drinks factory workers. Educational status, mixing department, working hours and service years of workers were statistically significantly associated with chronic respiratory symptoms. In this study, flour mill workers showed a statistically significant reduction in FVC (p < 0.002), FEV_1_ (p < 0.001) and FEV_1_/FVC (p < 0.012).

The development of at least one chronic respiratory symptom was higher among flour mill workers (56.6%) compared to the soft-drinks factory (12.86%). This study showed higher prevalence of chronic respiratory symptoms than in other flour mill factory studies, such as those in Ibadan, Nigeria (54% of production staff and 19% in controls), in Ilorin, North Central Nigeria (49.5% of the flour mill workers and 27.7% in controls) [26, 27].

The most common chronic respiratory symptoms in the study were chronic cough 39.3%, chronic breathlessness 18.9%, chronic cough with sputum 17.86% and chronic wheezing 17.35%. The result was higher than the study done in Addis Ababa, Ethiopia in flour mill workers, which was 27.7% dry cough, 14.8% wheeze, and 16.6% breathlessness [18].

On the other hand, the result of this study was lower than the study conducted in flour mill factories in Egypt that reported the prevalence of cough 87.5%, shortness of breath 60% and wheezes 72.5% [7]. The higher prevalence of chronic respiratory symptoms among flour mill workers might be because of lack of personal protective equipment and increased longer duration of exposure to flour mill dust compared to soft drink factory workers.

There is inadequate personal protective equipment in the work environment and the workers did not wear the personal protective equipment properly even if little equipment is available. This study finding is in line with studies conducted in India and Nigeria flour mill workers found that the most of the workers didn’t use personal protective equipment while working [6, 15, 28]. This might be because lack of proper training for workers or lack of awareness about the importance of personal protective equipment. So further study should be conducted to identify the reasons for not using PPE.

In this study, primary school educated workers were more likely at risk of developing chronic respiratory symptoms than workers who got secondary education and above. This result was in line with a study conducted in the Ethiopian factory [29]. Low educated workers might be assigned in risk job or it might be that they do not understand the risk of dust exposure so they might not take necessary precaution measures.

In this study, mixing department workers were more likely to develop chronic respiratory symptoms than workers in packaging, loading and cleaning departments. This finding was inconsistent with studies conducted in Iraq and Egypt, which reported that packaging department workers had a high prevalence of chronic respiratory symptoms compared to those of mixing, loading and cleaning department’s workers [7, 30]. This might be due to differences in working conditions like ventilation of working environment, machine type and use of the personal protective device.

This study showed that workers who had work experience 6–9 years and ≥ 10 years had the odds of developing chronic respiratory symptoms were 5.1 and 2.5 times more than workers who had work experiences between 1 and 5 years, respectively. This finding was in line with the studies conducted in Iraq and Egypt flour mill workers [7, 30]. This might be due to increased dust accumulation in the respiratory system associated with prolonged exposure to flour dust in the work environment. Also, working over 8 h per day was significantly associated with chronic respiratory symptoms. This finding was similar to a study conducted in Iraq flour mill workers [30]. This might be due to exposure for extended hours of increased dust accumulation in the respiratory system.

The present study’s results showed that among flour mill dust exposed workers a significant decline in the lung function parameters, FVC, FEV_1_ and FEV_1_/FVC compared to the soft-drinks factory workers. Meo et al., Dhillon et al., Meo and AL-drees and Melo et al. reported that flour mill workers had significantly reduced lung function parameters FVC and FEV_1_ [2, 15, 31]. However, in our findings, FVC, FEV_1_, and FEV_1_/FVC were significantly reduced among flour mill workers. This might be due to the difference in working conditions such as ventilation type, machine type and use of the protective device.

These study results were consistent with studies conducted by Mohammadien et al. and Deshpande et al., who observed that lung function parameters reduced in non-smoking flour processing workers and reported that the exposed group had significantly lower FVC, FEV_1_ and FEV_1_/FVC than the control group [7, 32]. In another study, Ige and Awoyemi investigated that the occupationally induced lung function impairment in bakery workers as a result of exposure to grain and flour dust. They reported that the mean values of FVC, FEV_1_ and FEV_1_/FVC in the bakery workers were significantly lower than those of the control subjects [12]. The reduction in FVC, FEV_1_ and FEV_1_/FVC among the flour mill workers might be due to increased dust accumulation in the respiratory system associated with prolonged exposure at workplaces.

## Limitations

One of the limitations of this study is healthy workers effect; workers who developed the symptoms may have quitted the job. The use of questionnaire method was also another limitation of this study because it may cause participants recall bias and interviewer bias. In addition, it would have been helpful if job designations for all workers were recorded and described in results since there may be specific types of workers at greater risk of respiratory symptoms. Furthermore, spirometry was not performed on all participants post-bronchodilator was not done in this study.

## Conclusions

Chronic respiratory symptoms were higher among flour mill factory workers than soft drink factory workers. Lower education level, increased work experience, work departments and longer working hours were determinant factors for the occurrence of chronic respiratory symptoms. The results showed that work-related exposure to a flour mill dust could reduce the mean values of lung function parameters such as FEV1, FVC, and FEV1/FVC. The study findings imply that there is a need to implement dust control methods to reduce workers’ exposure in the flour mill factory. Besides, a follow-up study with dust measurements on the effects of flour mill dust on respiratory health among flour mill production workers is recommended for the future.

## Data Availability

The datasets generated and/or analyzed during the current study are not publicly available due to study participant privacy/consent agreements but are available from the corresponding author on reasonable request.

## Abbreviations

ATS: American Thoracic Society
AOR: Adjusted Odds Ratio
BMRC: British Medical Research Council Questionnaire
CI: Confidence Interval
COR: Crude Odds Ratio
FEV_1_: Forced Expiratory Volume in one second
FVC: Forced Vital Capacity
FEV_1_/FVC: Ratio of Forced Expiratory Volume in 1 second and Forced Vital Capacity
SD: Standard Deviation
SPSS: Statistical Package for Social Sciences
SOPs: Standard Operative Procedures
